# Morbid Reflex Neurosis*Delivered before the Southern Surgical and Gynæcological Association and reported stenographically for the Southern Medical Record.

**Published:** 1892-04

**Authors:** Willis F. Westmoreland

**Affiliations:** Professor of Surgery, Atlanta Medical College, Atlanta, Ga.


### Southern Medical Record.

A MONTHLY JOURNAL OF MEDICINE AND SURGERY.

VOL. XXII. ATLANTA, GA., APRIL, 1892. NO. 4

### Original Articles.

### MORBID REFLEX NEUROSES.*

BY WILLIS F. WESTMORELAND, M. D.,
Professor of Surgery, Atlanta Medical College, Atlanta, Ga.

When we say *reflex neurosis* we mean nothing; that is a physiological condition. If it were not, you would not have a blush that a person has from any mental impression. I shall speak more particularly of those conditions where there is a *morbid reflex neurosis* where, from irritation at some particular point, you have a morbid reflex trouble. Take, for instance, the eye. As a result of some inequality in the ocular muscles you may have cerebral headache. Oculists go so far as to claim that you have epilepsy or chorea as a result of some inequality of the ocular muscles. Take, again, hypertrophy of the nose; as a result of that you may have acne of the face. As a result of dentition you may have convulsions in the child. All these are amenable to surgical treatment. You operate on the muscles of the eye, and remove the cause of the trouble. By removing the hypertrophy of the nose you relieve the condition. Take a case of asthma where a hypertrophy infringes upon the nasal space, and

*Delivered before the Southern Surgical and Gynæcological Association and reported stenographically for the Southern Medical Record.

by appropriate treatment you remove the cause. I deal with morbid reflex neuroses, as applied to the genito-urinary organs, in the same way.

We have two classes of stricture, viz., organic and spasmodic, or, for the sake of convenience, I will call the latter a functional stricture. It is a condition where there is no organic lesion except it be congenital. In some cases, however, you may have a narrowing of the meatus as a result of chancroid or some condition that will interfere with the space of the meatus. Now, wherever you have a contracted meatus, as a result of it you have a spasmodic stricture, only in those cases, however, where you have had some irritation or the patient is a masturbator. He has had gonorrhœa perhaps; he has had an excessively acid urine; in other words, as soon as there is some irritation of the urethra, no matter what the cause may be, he will have a spasmodic stricture, and as a result of it you have these morbid reflex neuroses. Every organ in the body can be affected.

Dr. Gross pointed out very early in his *Surgical Pathology*, more particularly as applied to women, that the uterus was connected with the stomach, the stomach with the lungs, the lungs with the heart, and the heart with the brain through the nervous system. The genito-urinary organs have the same nerve connections in men, though the morbid reflexes are not so frequent. Now, as a result of this condition, a patient comes to you with numerous symptoms, some of which I want particularly to point out.

A patient will come to you with many symptoms of disease, and you find that he has had no stricture, but owing to the indiscretions of youth he may have been a masturbator, although he has never had any venereal disease. Masturbation in a young subject, who has a large meatus, will not affect him like a boy who has a contracted meatus. As a rule they are born with a small meatus. Where you have a narrowing of the meatus, a man who masturbates or has any irritation will nearly always have a spasm, and as a result of that condition an impression is made upon the nervous system, which causes all of these reflex troubles. I will outline some of them. There is no particular part of the body that is not affected by these reflexes. It may appear in one patient here and another there. Only after you have seen a number of cases you will find that nearly every portion of the body has been affected. Agreat many patients come into my office and say they have Bright's disease; that they suffer intense pain in the back, have frequent micturition, especially at night. They nearly always have to get up during the night to pass urine. In nearly every one of these cases reaction of urine is normal. They not only frequently urinate at night, but they feel as thoug hthey would like to continue it, the bladder feeling as if it were not emptied of its contents.

Another class of cases come to you with heart trouble. They will say they have a nervous heart, one that is excessive in its action, and they are very much alarmed.

Another class of cases come to you where the trouble is entirely cerebral. They complain of having epileptic convulsions. They have all the phenomena of cerebral headache and inco-ordination; their memory is not as good as it was.

All of these symptoms show that an impression has been made upon the brain and nervous system.

Again, you may have patients—as I have had them—coming to you with four or six convulsions within twenty-four hours. You may have an intermission or continuation of that proportion. I operated on one patient who had on an average, three or four convulsions every three months. You find other patients with acne of the testicles, with an intense itching which is difficult to control. There is really no disease of the skin itself. By continually grasping and rubbing the testicles between the hands the itching is somewhat increased. We find fissure in ano as a result of this form of stricture. In short, we have all of those various troubles, in some patients so marked as to drive them almost to insanity. I have seen patients, who, if they had been put on a witness-stand and tried for insanity, would have been sent to an insane asylum—in fact, the principal physician of an insane asylum told me that he had six cases within the last year or two, in which there was no doubt in his mind but what their troubles were due to this condition. Two of them were relieved by operation. Sometimes you have the whole system affected by it. I had a patient in college who had not walked for two years without the aid of crutches. He was confined to his bed part of the time, his condition simply being due to spasmodic stricture. After section of the meatus and the introduction of a full-sized bougie, in three weeks thereafter he walked before the class without crutches. We are too prone to overlook this condition. In cases in which you can pass a No. 23 or 24 sound French scale, you may think the man's urethra is normal; that he has no stricture, and very few symptoms of it. Let me say right here, that one of the worst cases of morbid reflex neurosis I have ever had, was a patient in whose urethra I could introduce a No. 30 acorn sound, and after section of the meatus up to 33 and introducing a bougie of that size, he was relieved of all symptoms. I simply report these cases to impress upon you that a narrowing or contraction of a few millimetres is sometimes sufficient to cause trouble. In this class of patients, the plan I follow is to operate upon them. First to enlarge the meatus, then begin the introduction of a full-sized sound. You have patients come to you who have nightly emissions, perhaps two or three emissions nightly. The mere sight of a woman will cause an emission. Dr. Griggs told me last night of a case he had had, where a man had gone to a circus, and the mere sight of a woman dressed in tights caused him to have a convulsion, and he fell off his seat and came near killing himself. In such cases we should go further than simply treat the spasmodic stricture by the mechanical process of introducing sounds. We should control their thoughts, put them upon hygienic treatment and a good general tonic. In addition to that, if their thoughts are always dwelling upon sexual matters, I generally control them by having them commit a piece of poetry or prose to memory, and by repeating this when these thoughts begin to occur, they control the perversion of the mind toward sexual ideas. Whenever I have patients of this kind I put them in a gymnasium and make them go to work. I do not think there is anything that controls the perversion of the mind like muscular exercise. The fatigue and improved nervous condition in a patient as a result of exercise of this kind, influence very materially the nervous conditions resulting from spasmodic strictures.

An excessive elongation of the prepuce frequently causes this trouble, particularly so if adherent. My experience with club-feet has been, that you rarely ever see trouble in girls. In the majority of cases, you find it in boys, and as a rule, you will find the majority of those boys have trouble with their genito-urinary organs. The boy either has an adherent prepuce, one that is very much contracted, or it is an excessively long one. Some of the worst troubles that have come under my observation was where the prepuce could be retracted but was elongated and very tight, and where all of the nervous symptoms were relieved by the operation of circumcision. In these cases we have other complications. We frequently have varicocele or excessive elongation of the scrotum, and conditions of that kind. You operate on your patient for stricture or perform circumcision and the nervous symptoms are kept up. In two or three cases of this kind I have been able to control them by grappling with this elongation of the scrotum. You will find that a great many of these cases have varicocele or elongation of the scrotum.

In another class of cases, you will find, after you have operated upon them, relief of symptoms for awhile, then the old troubles begin anew. Upon examination of the man's rectum you will find that he has fissure, and when that fissure is stretched you get permanent relief of all symptoms which were not relieved by the operation for stricture.

Another class of cases you have to deal with are cases of fissure in ano. Of course, this is applicable to the female as well as the male. In these cases you have all the reflex neuroses, as a rule, applied to the abdomen in women. You have pain all through the belly; you have constipation; the patient does not have an action of the bowels without taking something to act upon them. A great many cases of chronic constipation are due to fissure which may not be well developed. In speaking of fissure, I would say that fissure is a misnomer; it is nothing more than a spasmodic contraction of the sphincter ani. Patients have the symptoms without the presence of an ulcer. By a fissure, we mean as a rule, we have an ulcer there, and as a result of the irritation produced by it, you have a spasmodic contraction of the muscles. I want to place myself on record as saying, that in the majority of cases I have seen there has been no ulcer, nor has there been any evidence of its being present in the rectum at the time of examination and operation. They are frequently due to other causes, and those causes are too numerous to mention. I have not time to go into details.

Where you have a spasmodic condition, you frequently establish in the female engorgement of the womb, the trouble perhaps being referred to the vagina or uterus entirely. I have operated on several in the female where the patients had been in the hands of gynecologists for years, and had been treated for uterine trouble. These patients entirely recovered simply by stretching the sphincter muscle. There is one gentleman in the room who has seen some of these cases with me.

I wish to speak now with reference to operations for fissure in ano. A great many physicians recommend the use of speculums for stretching the muscle; while other operators advise you to take a knife and nick the muscle before you stretch it. In olden times surgeons introduced their hands into the rectum, closed their fist and then pulled it out, stretching the muscle in that way. In the use of these different methods you cannot tell how far you stretch the muscle. I have never seen patients lose control of the sphincter as a result of this operation where the fingers or thumb had been used as a method of operation. Incontinence of the feces occurs in those cases where the muscle has been cut, stretched with speculum, or where some of the old methods have been used. My plan is to put the patient under the influence of an anæsthetic. I stretch the sphincter; I feel the contracted parts of it have given way while my patient is under the influence of an anæsthetic; I take my fingers out, and then if there is not any contraction of the muscle--none at all--I let it alone. If the sphincter contracts, it shows that the contracted part is not thoroughly broken up, and I stretch it more while the patient is still under the influence of the anæsthetic.

REMARKS.—I will try and answer the different questions that have been brought out in their regular order: First, in reference to the point made respecting fissure in ano so called. That is one of the principal points that I wish to impress upon your minds. This condition, in my experience, has resulted from other conditions as frequently as it has from an ulcer; in other words, that it is a spasmodic stricture or contracture of the sphincter muscle. I like the word Sayre uses: the distinction between contracted and contractured muscle, and if I may apply this distinction, I should say it was a contracture of the muscle in the majority of cases. Of course, if you had a fissure of the anus the result of an ulcer, it could not be connected with the stricture; it might cause spasmodic stricture; it might be a source of irritation. The point I desire to impress upon you is this: Allingham has said that in the majority of cases fissure in ano is the result of ulceration, a crack or fissure which occurs between the folds of the muscle; that you nearly always have a teat as the result of it. You can run your fingers up behind that teat and find an ulcer above it. In the majority of cases that I have seen there has been no condition of that kind. In a great many instances, you have a spasmodic contraction of the muscle without previously having had an ulcer. When I see a case of this kind, and symptoms of stricture have commenced to appear, before the patient has had any pain or suffered with his bowels, I conclude that it is a reflex which has caused that contraction of the sphincter, and the contraction of the muscle will disappear to a certain extent if you operate on the stricture. I have in one or two cases found a powerful contraction of the muscle where I operated perhaps before the fissure in ano occurred, and the patient got well. The spasmodic contraction had ceased with the improvement of the case.

In reference to elongation of the prepuce causing these troubles, I am sure that it does. When I see a patient with a morbid reflex of this kind, symptoms very much exaggerated, I do not do anything except circumcise him; then the symptoms rapidly disappear. If the patient gets well as a result of the operation, I naturally attribute his trouble to that cause.

I have never done the operation of the Jews, consequently

I do not know of some of them being ruined by their method of circumcision. If I had my way I would circumcise every boy that came into the world. The best time to do circumcision is when they are from one to eighteen months old. I circumcise every child that comes within the ramification of my family practice, and expect to keep it up.

With regard to the point that you frequently find a contracted meatus in men who were circumcised when children, I will say my experience, in the majority of cases, is that where you have a tightly adherent prepuce, you have a contracted meatus, and that in a large proportion of the cases you operate on for that cause you make both operations before the child entirely recovers. You not only circumcise him, but you make section of the meatus. My experience has been in the majority of cases of tight, adherent prepuce, that you nearly always have this contraction of the meatus, and I usually, in these cases, have to do both operations before the patient recovers. I have seen patients who had been circumcised perfectly, yet with all the nervous symptoms they had before the operation. The operation did them no good. Whenever a patient of that kind comes under my observation, I find that he has a contracted meatus as well, and I operate upon it.

These cases are frequently mistaken for organic stricture. Very frequently you will have a patient come to you who is certain that there is organic stricture. In such a case I cut the meatus and the symptoms of organic stricture, caused by sensitive points along the urethra, disappear. If the patient's meatus or urethra will measure No. 32, I cut it to 33. I cut it at least one millimetre larger than the size of the instrument I expect to introduce. I do that for the reason that frequently you have irritation as a result of the operation. In the union of the wound you have a contraction of the fibrous tissue which generally reduces the calibre of the meatus one millimetre. If you cut it to 32 and continue to use a 32 instrument, you have allowed absolutely no room for the contraction of the cicatrix. The result is that while your instrument goes in, you will always have more or less irritation following the introduction—that has been my experience. I have never seen a man cut, no matter to what size, whose urethra did not close up to the size of the instrument that was introduced. This idea of having cut a man so large that he splatters all over the place is all foolishness. The trouble is the canal closes too rapidly, and we may have to make another section. I have had several cases of this kind. Very frequently I have thought I had an organic stricture, and have promptly cut the meatus, after which I found this contractility which seemed to follow the whole urethra, even the penile portion, has disappeared. After section of the meatus it disappears entirely, and I am enabled to introduce an instrument without any trouble.

Another point. I want to say that my experience is, when a man has cystic trouble as a result of this operation, it is the fault of the surgeon who uses the bougie as a rule; that the irritation comes from the way the bougie was introduced, and does not arise from the fact that the mechanical introduction of it will cause vesical irritation or cystitis as a sequelæ. You have got a very delicate mucous membrane there. When you attack that delicate mucous membrane you should use vaseline on your instrument. There is enough adhesion between the delicate epithelial layers and the bougie, if you carry your instrument in rapidly, to cause disturbance and irritation of the whole canal, especially in the neck of the bladder, where the point that you turn rapidly infringes against the upper portion. Very frequently you have pockets in that portion of the urethra where, if you are not exceedingly careful in introducing it and carry it down with the slightest degree of force, it is enough to set up irritation of that portion of the urethra. I take an instrument, and, if necessary, take five minutes to put it in. When I commenced practice, I introduced my instruments rapidly, and in almost every case in which I introduced it in this way I had either incontinence of urine or considerable irritation; consequently I have since been more careful, and exerted every degree of patience and skill to go slow in introducing the instrument. Then, again, a great many physicians, although they may be careful in the introduction of the instrument, will pull it out with a great flourish, which, in my opinion, causes as much irritation as it does in putting it in. You will find some patients in which a long curved instrument will not go in without the exertion of considerable force; whereas, by using an Otis instrument, it will go in without trouble, owing to some abnormal outline of the urethra. I frequently see these cases. I keep instruments with different curves. If I have trouble in introducing one, I substitute another, and usually can select one that will go in with comparative ease.

With regard to the point that a contracted meatus is apt to occur as a result of some venereal trouble or lesion, congenital or acquired, I think it very rare. In a practice extending over six years in this particular line, I have only seen two cases. It is unusual for a chancroid or chancre to occur in a meatus, or borders of it, so as to cause contraction as a result of the cicatrix or destruction of tissue. In these cases there were no unpleasant sequelæ, because, as soon as the chancre got well in one case, I cut the meatus to full size. In the other case, where it occurred eight or ten years before, I had all the reflexes you would have from a regular spasmodic stricture.

